# EVAR Trends over the Past Decade and Their Impact on Aneurysm Mortality: National Health Insurance Data Analysis

**DOI:** 10.3390/jcm14155277

**Published:** 2025-07-25

**Authors:** Sungsin Cho, Jin Hyun Joh

**Affiliations:** Department of Surgery, Kyung Hee University Hospital at Gangdong, Seoul 05278, Republic of Korea; 01ssunny@naver.com

**Keywords:** aortic aneurysm, abdominal, mortality, endovascular aneurysm repair, open abdomen techniques

## Abstract

**Background/Objectives**: There are no reports about the nationwide trends in abdominal aortic aneurysm (AAA) repair and mortality rates. This study aims to evaluate the trend in AAA treatment and related mortality, including ruptured AAAs (rAAAs) and intact AAAs (iAAAs) over the last 13 years. **Methods**: This serial, cross-sectional study investigated the time trends in patients who were treated for an AAA and underwent an aneurysm repair between 2010 and 2022. Data from the Health Insurance Review and Assessment Service (HIRA) and Statistics Korea were used. A linear-by-linear association and Poisson regression analysis were performed to determine the changes in the treatment of AAAs and related mortality. **Results**: The number of patients with an rAAA increased from 462 in 2010 to 770 in 2022 (relative risk, RR 1.57; *p* < 0.0001). The number of patients with an iAAA increased from 3685 to 12,399 in the same period (RR 3.16; *p* < 0.0001). Endovascular aneurysm repair (EVAR) has been more commonly performed since 2011. During the study period, EVAR increased from 406 to 1161 (RR 2.68; *p* < 0.0001). Although the annual mortality rates after iAAA treatment decreased from 1.4% to 0.7% (mean mortality rate, 1.1%), the mortality rates after rAAA treatment were similar, ranging from 34.6% to 34.2%, during the study period (mean mortality rate, 35.2%). **Conclusions**: During the last 13 years, the annual number of patients with rAAAs and iAAAs has increased. Since 2011, EVAR has been more commonly performed. The annual iAAA-related mortality rate decreased along with the increasing trend in EVAR. However, the annual rAAA-related mortality rate did not change.

## 1. Introduction

Abdominal aortic aneurysm (AAA) is an aging-related disease, and the larger the size, the higher the risk of rupture. The endovascular aneurysm repair (EVAR) was first introduced in 1991 as a minimally invasive vascular repair [1]. Since then, numerous studies have demonstrated the benefits of EVAR compared with conventional open aneurysm repair (OAR). Among them, three significant randomized controlled trials consistently reported that EVAR resulted in a significant reduction in 30-day mortality [2,3,4]. Because EVAR was reported to reduce 30-day mortality dramatically, there was a significant reduction in the number of OARs subsequently.

These reports led to a shift in the concept of an optimal treatment strategy for patients with an AAA. With a dramatic increase in the number of EVARs, several guidelines have reflected this change in concept. Representatively, the Society for Vascular Surgery (SVS) guidelines emphasized the benefits of EVAR for perioperative mortality and recommended it as the preferred treatment option for anatomically fit AAA patients with a 10-year life expectancy [5]. The European Society for Vascular Surgery (ESVS) guidelines proposed the same recommendations [6]. Therefore, EVAR is considered the primary modality to treat AAA patients who have an anatomically suitable AAA.

There is a lack of comprehensive, nationwide data assessing the trends in AAA repair techniques and their effects on mortality rates, particularly for both ruptured AAA (rAAA) and intact AAA (iAAA) cases. Existing research and clinical guidelines increasingly favor EVAR over traditional OAR due to its minimally invasive nature. However, the long-term impact of EVAR on aneurysm-related mortality, especially for rAAA, remains unclear. Thus, there is a need to evaluate the impact of the increasing use of EVAR compared to OAR on aneurysm-related mortality across an entire population. Therefore, the purpose of this study was to evaluate the nationwide trends in annual AAA treatment and aneurysm-related mortality, including rAAAs and iAAAs over the past 13 years.

## 2. Materials and Methods

The data source for the recent 13-year-period analysis was the Health Insurance Review and Assessment Service (HIRA), comprising data from 2010 to 2022. Korea’s public medical insurance system covers almost the entire population of Korea. HIRA is a government-operated organization, and healthcare service providers submit claims data to HIRA for reimbursement. Therefore, the HIRA data are national characteristic data for actual patients in Korea [7]. The HIRA Data Access Committee and Kyung Hee University Hospital at Gangdong Institutional Review Board (IRB no. KHNMC 2015-01-027) approved the use of their data in this study. Kyung Hee University Hospital at Gangdong Institutional Review Board has waived informed consent for this study. All methods were carried out using relevant guidelines, including RECORD guidelines and regulations.

The numbers of patients with AAAs were collected using the Korean Standard Classification of Diseases and Causes of Death (KCD-7), as this complementary system is an adaptation of the International Statistical Classification of Diseases and Related Health Problems, 10th Revision (ICD-10), to Korean conditions. The codes of I713 and I714 represent rAAAs and iAAAs, respectively. The annual numbers of OAR and EVAR were collected using the Electronic Data Interchange (EDI) codes. The open repairs for AAAs were classified with O0223, O0224, and O2034 for suprarenal AAA, infrarenal AAA, and abdominal aorta involving the iliac artery, respectively. The EDI code for EVAR is allocated with only one code of M6612. The detailed KCD and EDI codes for rAAA, iAAA, and AAA repairs are shown in Table 1.

The annual AAA-related mortality rate was collected using Statistics Korea. The MicroData Integrated Service (MDIS) of Statistics Korea releases the cause of death due to specific diseases. The causes of death were classified according to the recommendations of the World Health Organization (WHO). The annual number of patient deaths with rAAAs and iAAAs was obtained from the server of MDIS. Then, the annual morality rate due to rAAAs and iAAAs was calculated as the percentage of deaths among the total number of patients with rAAAs and iAAAs.

For statistical analysis, a linear-by-linear association was performed to determine trends in AAA patients and treatments for this period. The Bland–Altman method was used to evaluate trends in population-adjusted frequencies. We measured the relative risk to evaluate trends using MedCalc Statistical software (Version 23.3.2, Ostend, Belgium). The annual trend in mortality rate was analyzed with Poisson regression analysis using the SPSS version 22.0 statistical software (SPSS Inc., Chicago, IL, USA). A *p*-value less than 0.05 (two-sided) was considered statistically significant.

## 3. Results

### 3.1. Annual Numbers of rAAAs and iAAAs

**Figure 1:**
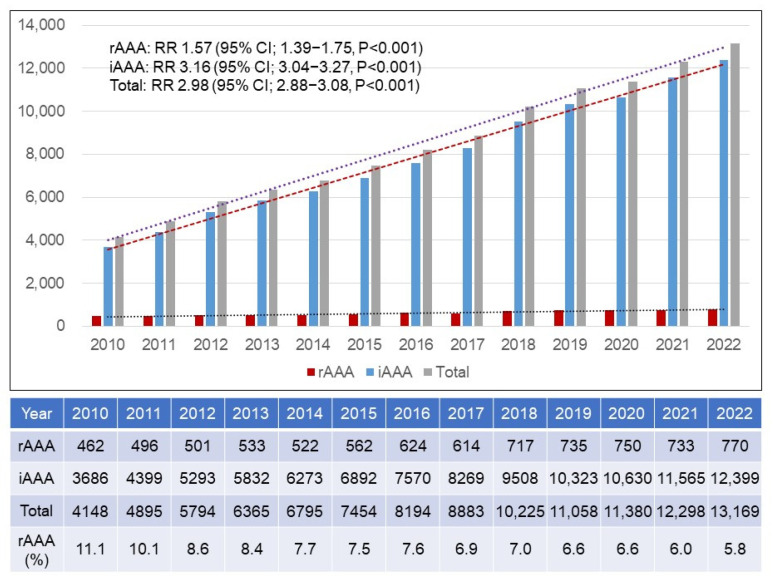
Annual number of patients who were treated with ruptured and intact abdominal aortic aneurysm repairs. rAAA = ruptured abdominal aortic aneurysm; iAAA = intact abdominal aortic aneurysm; RR = relative risk; CI = confidence interval.

There was a total of 110,658 patients with AAAs from 2010 to 2022, of which 8019 had an rAAA while 102,639 had an iAAA. Figure 1 represents the annual number of patients with an AAA. The annual number of rAAA patients increased from 462 in 2010 to 770 in 2022 (relative risk, RR 1.57; 95% confidence interval, CI 1.39–1.75; *p* < 0.001). Compared to rAAAs, the annual number of iAAAs increased more rapidly from 3686 in 2010 to 12,399 in 2022 (RR 3.16; 95% CI 3.04–3.27; *p* < 0.001). The rapid increase in the number of iAAA patients resulted in a 2.98-fold increase in the total number of AAA patients during the study period (RR 2.98; 95% CI 2.88–3.08; *p* < 0.001).

### 3.2. Gender Distribution

**Figure 2:**
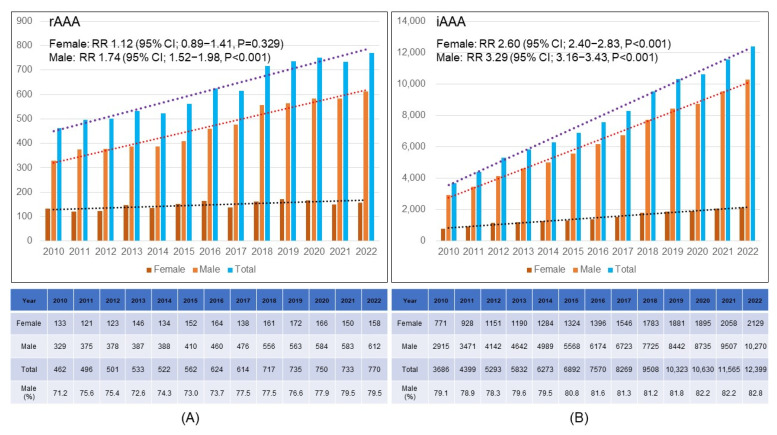
**Annual distribution of the number of patients with abdominal aortic aneurysm by gender. (A) Ruptured AAA, (B) Intact AAA.** rAAA = ruptured abdominal aortic aneurysm; iAAA = intact abdominal aortic aneurysm; RR = relative risk; CI = confidence interval.

During the study period, the annual number of male rAAA patients increased gradually (RR 1.74; 95% CI 1.52–1.98; *p* < 0.001), while that of female rAAA patients showed no statistically significant increase (RR 1.12; 95% CI 0.89–1.41; *p* = 0.329). Therefore, the proportion of male rAAA patients increased from 71.2% in 2010 to 79.5% in 2022 (Figure 2A). Figure 2B demonstrates the annual changes in gender distribution of iAAA patients. The annual numbers of male and female iAAA patients increased 3.29-fold and 2.60-fold, with statistical significance (*p* < 0.001).

### 3.3. Age Distribution

**Figure 3:**
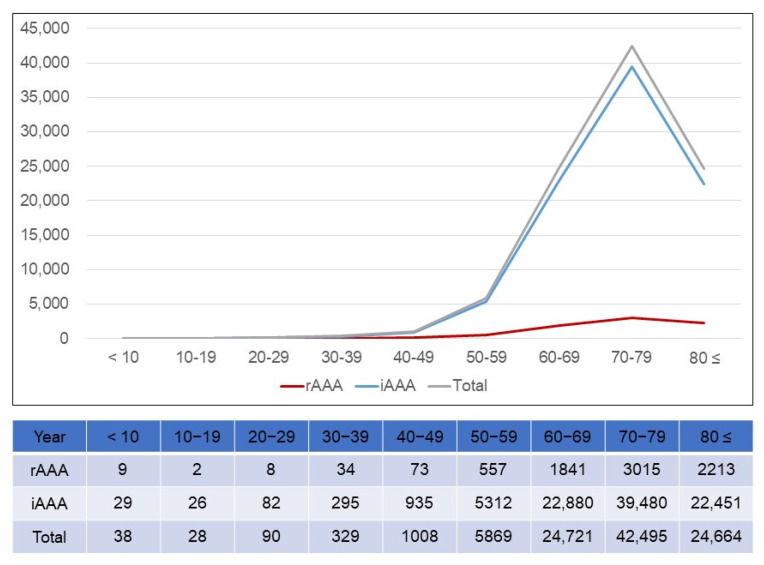
Distribution of number of patients with abdominal aortic aneurysm by age. rAAA = ruptured abdominal aortic aneurysm; iAAA = intact abdominal aortic aneurysm.

The patients most frequently treated for AAAs were those in their 70s, regardless of the presence of a rupture (Figure 3). In the rAAA group, a rapid increase was shown in individuals in their 60s and a peak was demonstrated in individuals in their 70s. However, in the iAAA group, a rapid increase was shown in individuals in their 50s. This was 10 years younger than the rAAA group. A more rapid increase was shown in individuals in their 70s in the iAAA group. In the rAAA group, individuals in their 80s and older ranked second followed by those in their 60s. In the iAAA group, those in their 60s ranked second, followed by those in their 80s or older. The annual number of AAA patients over 70 years was significantly increased from 2374 (57.2%) in 2010 to 9594 (72.9%) in 2022 (RR 4.04; 95% CI 3.87–4.22; *p* < 0.001). During the same period, the number of octogenarian AAA patients tended to increase statistically significantly to 206 (5.0%) in 2010 and 1104 (8.4%) in 2022 (RR 5.36; 95% CI 4.63–6.19; *p* < 0.001).

### 3.4. Annual Trends in EVAR Versus OAR

**Figure 4:**
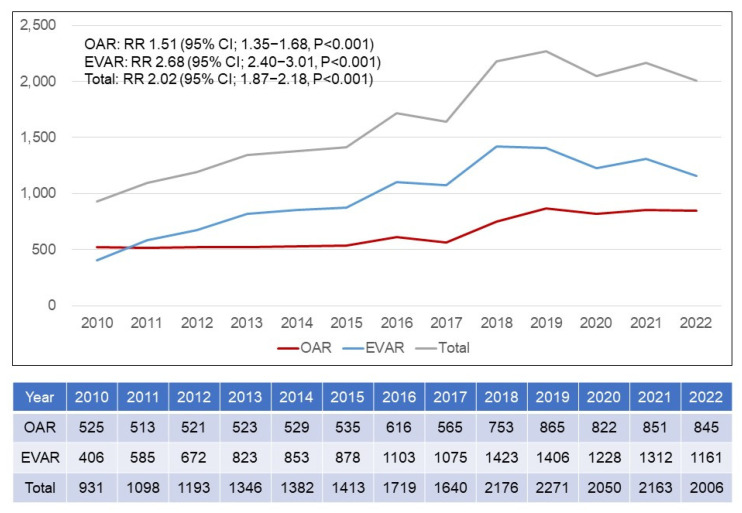
**Annual number of patients who underwent open and endovascular repair of an abdominal aortic aneurysm.** OAR = open abdominal aortic aneurysm repair; EVAR = endovascular abdominal aortic aneurysm repair; RR = relative risk; CI = confidence interval.

Figure 4 shows the annual number of patients who underwent an aneurysm repair by either an open repair or endovascular repair. The total number of aneurysm repairs increased significantly from 931 in 2010 to 2006 in 2022 (RR 2.02; 95% CI 1.87–2.18; *p* < 0.001). During the 13-year period, the annual number of patients who underwent OAR increased from 525 in 2010 to 845 in 2022 (RR 1.51; 95% CI 1.35–1.68; *p* < 0.001), with a gradual upward trend. However, the number of patients who underwent an EVAR markedly increased from 406 in 2010 to 1161 in 2022 (RR 2.68, 95% CI 2.40–3.01, *p* < 0.001). In 2010 and 2011, the number of patients who underwent an EVAR annually surpassed those who underwent an OAR. As the years passed, the number of patients who underwent an EVAR was higher than that of those who underwent an OAR. Table 2 demonstrates the annual trends in OARs by anatomic level, gender, and octogenarians. The proportion of suprarenal OAR was not changed, with an average of 15.9%. The number of OAR in the male group markedly increased from 426 in 2010 to 728 in 2022 (RR 1.71; 95% CI 1.52–1.92; *p* < 0.001). Interestingly, however, there was no statistically significant change in the female group. In addition, the number of OARs in octogenarians showed a statistically significant increase from 48 (9.1%) in 2010 to 146 (17.3%) in 2022 (RR 3.04; 95% CI 2.19–4.21; *p* < 0.001). During the same period, the number of EVARs in octogenarians significantly increased from 59 (14.5%) in 2010 to 358 (30.8%) in 2022 (RR 6.07; 95% CI 4.61–7.98; *p* < 0.001).

### 3.5. Mortality Rate

**Figure 5:**
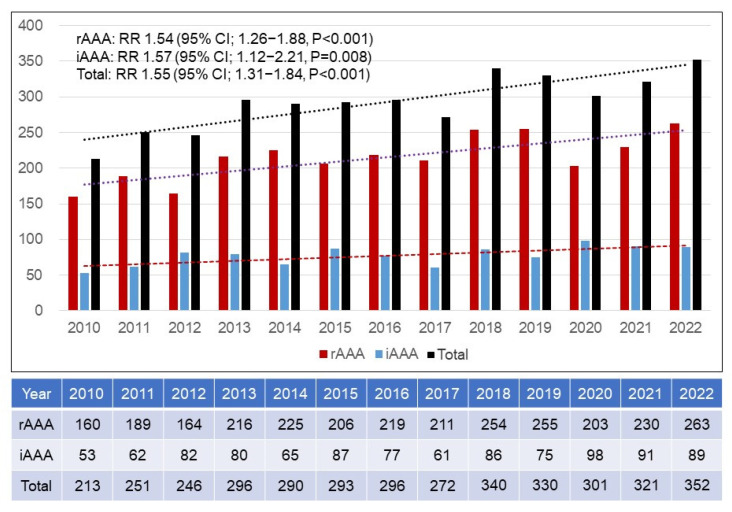
Annual number of patients who died of ruptured and intact abdominal aortic aneurysms. rAAA = ruptured abdominal aortic aneurysm; iAAA = intact abdominal aortic aneurysm; RR = relative risk; CI = confidence interval.

**Figure 6:**
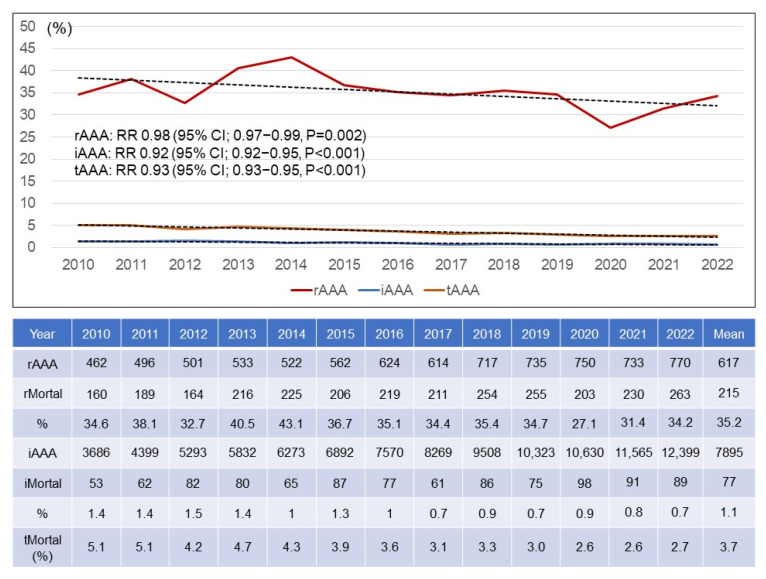
**Annual trends of mortality rate due to ruptured and intact abdominal aortic aneurysms.** rAAA = ruptured abdominal aortic aneurysm; iAAA = intact abdominal aortic aneurysm; tAAA = total abdominal aortic aneurysm; RR = relative risk; CI = confidence interval.

Figure 5 demonstrates the annual numbers of patients who died with an rAAA or iAAA. Total numbers of patients with rAAAs and iAAAs increased gradually by 1.54-fold and 1.57-fold, respectively. Therefore, the total number of died patients with an AAA slowly increased from 213 in 2010 to 352 in 2022 by 1.55-fold (RR 1.55; 95% CI 1.31–1.84; *p* < 0.001). Figure 6 shows the total numbers of patients and patients who died with rAAAs and iAAAs. With those numbers, the annual morality rates of AAAs were calculated. The morality rate of rAAAs was 34.6% in 2010 and 34.2% in 2022 with no changes. Although the total number of patients who underwent an EVAR surpassed that of those underwent an OAR as of 2010 and 2011, the annual mortality rates were similar with an average mortality rate of 35.2%. However, the annual mortality rates of iAAAs were 1.4% in 2010 and 0.7% in 2022, with a downward trend. The mean mortality rate of iAAAs was 1.1%.

## 4. Discussion

In this study, AAAs nearly tripled over the past 13 years, mainly due to an increase in iAAAs. In the annual distribution by gender, iAAAs increased in both men and women, but rAAAs increased in men, while there was no statistically significant increase in women. Looking at the distribution by age, both rAAAs and iAAAs were the most prevalent in individuals in their 70s. With the increase in AAAs, OAR and EVAR showed an increasing trend, and EVAR surpassed OAR in 2011. Over the past 13 years, the mortality rate of iAAAs has decreased, while the rate of rAAAs has not changed significantly.

It is presumed that the increase in AAAs, including rAAAs and iAAAs, is largely due to health service examination. The diagnosis rate of AAAs increased due to the use of abdominal ultrasound, which was included in the health service examination and was widely performed. The introduction of a screening program for AAAs has been contemplated by health services in several countries [8]. Many studies demonstrated that AAA screening lowered the mortality rate from AAAs and provided benefits in terms of cost-effectiveness. The UK Multicentre Aneurysm Screening Study (MASS) randomized trial showed that screening resulted in a reduction in all-cause mortality, and the benefit in AAA-related mortality continued to accumulate throughout follow-up [9]. In addition, the U.S. Preventive Services Task Force reported that a one-time invitation for AAA screening in men aged 65 years or older was associated with decreased AAA rupture and AAA-related mortality rates [10]. A systematic review on the long-term benefits of one-time AAA screening revealed that population-based one-time screening for AAAs with ultrasound in asymptomatic men aged 65 years and older remains beneficial during the longer term after screening has ceased, with significant reductions in AAA mortality and AAA rupture rates, and hence avoids unnecessary AAA-related deaths [11]. Another reason was that the departments treating AAAs have diversified. Previously, OAR and EVAR were performed in vascular surgery and interventional radiology, respectively. But now, AAA can be treated in general surgery and cardiothoracic surgery, as well as in interventional cardiology, in addition to the previous two departments [12]. Another important reason for the increase in AAAs might be the increase in public awareness. Tilson MD insisted that AAAs are a silent killer and we need to raise public awareness of this deadly disease [13].

Interesting results could be found by looking at the annual distribution of rAAAs and iAAAs by sex. In iAAAs, they increased in both men and women, whereas in rAAAs, they increased 1.74-fold in men, but there was no significant change in women over the past 13 years. This was speculated to be due to the high frequency of rAAAs in men overall. Another reason might be the probability of rAAA repair by sex. Malayala SV compared the characteristics of rAAAs in females and males [14]. The probability of undergoing surgery for an rAAA was significantly lower for females as compared to males (*p* = 0.03). However, we should consider the important finding that women tended to have a ruptured aneurysm of a smaller size, faster growth, a higher frequency of rupture, and a higher mortality rate, though the overall incidence of AAA rupture was higher in males [14,15].

According to the distribution of AAAs by age, AAAs increased as age increased until individuals reached their 70s and then decreased in their 80s. Singh K et al. assessed the age- and sex-specific distribution of the abdominal aortic diameter and the prevalence of and risk factors for AAAs [16]. The prevalence of AAAs increased with age. The mean aortic diameter increased with age in both men and women (*p* < 0.001), although the increase was more pronounced in men. From the age of 55 years, there was a pronounced increase in both standard deviation and skewness, particularly in men. Howard DPJ et al. reported population-based data on the age-specific incidence of acute AAAs [17]. The incidence per 100,000 individuals per year was 55 in men aged 65–74 years, but increased to 112 at ages 75–84 years and to 298 at ages of 85 years or above. Figure 3 showed that although AAAs were most prevalent in individuals in their 70s, iAAAs showed a rapid increase in individuals in their 40s, whereas rAAAs showed a rapid increase in individuals in their 50s, 10 years later. Although this study did not show a detailed age-dependent distribution of rAAAs and iAAAs, it showed that the rapidly increasing age for rAAAs was higher than the rapidly increasing age for iAAAs. Gormley S et al. identified that the mean ages of iAAA and rAAA occurrence were 75.1 years and 77.8 years, respectively [18]. Studies in other countries also showed a trend of increasing frequencies of AAAs as age increases, as in this study.

This nationwide data analysis showed that the number of EVAR procedures increased from 406 in 2010 to 1161 in 2022, with a 2.68-fold increase. The EVAR procedure has the advantage of being less invasive than a standard OAR, which has been performed with the basic concept of aneurysm exclusion. Since EVAR is a minimally invasive procedure for AAA patients, the increase in the number of procedures was expected to result in improvements in procedural outcomes, such as aneurysm-related death. However, there were studies showing contradictory results in the long term. Yei K et al. reported the long-term outcomes associated with EVAR compared with OAR using the Medicare-matched database [19]. Overall mortality after an elective AAA repair was higher with an EVAR than OAR, despite reduced 30-day mortality and perioperative morbidity after an EVAR. Endovascular repair was additionally associated with significantly higher rates of long-term rupture and reintervention. Also, the UK EndoVascular Aneurysm Repair (EVAR) randomized controlled trial showed that EVAR had an early survival benefit, but an inferior late survival benefit compared with OAR [20]. However, Lederle FA demonstrated that long-term overall survival was similar among patients who underwent an EVAR and those who underwent an OAR. A difference between groups was noted in the number of patients who underwent secondary therapeutic procedures [21].

With the rapid increase in EVAR over the past 13 years, the iAAA mortality rate decreased from 1.4% in 2010 to 0.7% in 2022, while the rAAA mortality rate did not change. In this study, we could not investigate the detailed post-EVAR and post-OAR mortality for rAAAs due to the use of public data. A systematic review and meta-analysis addressed mortality after repair of an rAAA [22]. In-hospital and/or 30-day mortality ranged between 0% and 54% in different series, whereas the pooled mortality after EVAR was 24.5%. In addition, with the mortality after both endovascular and concurrent open repair from the same unit, the pooled mortality after OAR was 44.4%. The pooled overall mortality for rAAAs undergoing an endovascular or open repair was 35%, showing a similar result to our study. Hoornweg LL et al. reported changes in mortality after rAAA repairs over time [23]. Mortality after an rAAA repair has not changed over the past 15 years. They assumed that this could be explained by the increased age of patients undergoing an rAAA repair.

A more in-depth investigation is required to understand the factors contributing to the persistent high mortality rate associated with rAAAs. The analysis should include factors such as the volume of cases performed at specific centers, the use of standard versus non-standard EVAR techniques, and the employment of specialized devices to accommodate complex anatomies. Given the concerning lack of improvement in rAAA mortality, it is critical to develop and implement standardized protocols and best practices for the management of rAAA patients. Such protocols could encompass aspects of pre-operative resuscitation, surgical technique, and post-operative care, tailored to the specific challenges posed by rAAA.

The results of this study are expected to contribute to the recognition of the need to make a protocol that supports policies promoting EVAR for suitable iAAA candidates and lowers the mortality rate of rAAAs by establishing the national health care policies in the future. The observed increase in AAA diagnoses, especially iAAAs, underscores the importance of targeted screening programs, particularly for individuals with risk factors such as age, smoking history, and a family history of aneurysms [16]. Identifying and treating iAAAs before rupture remains a crucial strategy for reducing overall AAA-related mortality. This study highlights the ongoing need to increase public awareness about AAAs as a potentially life-threatening condition. Increased awareness can encourage at-risk individuals to seek screening and prompt, timely intervention, ultimately reducing the incidence of rupture and improving outcomes.

This study has several limitations. First, this is a retrospective study based on an administrative database. Therefore, there was an intrinsic limit to the number of variables that could be measured. Second, the purpose of the database is to collect the patient and procedure numbers only. Third, follow-up data and long-term outcomes were unavailable. Finally, there was no detailed post-EVAR and post-OAR mortality for rAAAs and iAAAs due to the use of public data. Further evaluation is needed after more detailed data are collected, including those specifying the center volume, the application of standard or non-standard EVAR, and the use of an iliac branch device.

## 5. Conclusions

The number of patients with AAAs has increased during the last 13 years. With the increasing trend in the number of EVAR procedures, the mortality rate associated with iAAAs decreased by 50%, while the mortality rate associated with rAAAs showed no significant change, averaging 35.2%.

## Figures and Tables

**Table 1:** Disease and procedure codes for abdominal aortic aneurysm.

KCD codes ^1^	Diseases
I713	Abdominal aortic aneurysm, ruptured
I714	Abdominal aortic aneurysm, without mention of rupture
EDI codes ^2^	Open repair
O0223	Abdominal aorta, suprarenal
O0224	Abdominal aorta, infrarenal
O2034	Abdominal aorta involving iliac artery
	Endovascular repair
M6612	Placement of stent graft, abdominal aorta involving iliac artery

KCD = Korean standard classification; EDI = Electronic Data Interchange. ^1^ KCD code is the Korean Standard Classification of Diseases and Cause of Death, which is classified by recommendation of the International Classification of Disease and Cause of Death (ICD). ^2^ EDI database from Health Insurance Review and Assessment Service (HIRA) is the classification for the operation/management and diagnosis.

**Table 2:** Annual trends in open abdominal aortic aneurysm repair by anatomic level, gender, and octogenarians.

Year	2010	2011	2012	2013	2014	2015	2016	2017	2018	2019	2020	2021	2022
Total number	525	513	521	523	529	535	616	565	753	865	822	851	845
Suprarenal	454	422	454	437	442	461	521	456	631	702	697	713	721
Infrarenal	71	91	67	86	87	74	95	109	122	163	125	138	124
Suprarenal %	13.5	17.7	12.9	16.4	16.4	13.8	15.4	19.3	16.2	18.8	15.2	16.2	14.7
Male	426	423	429	421	437	421	492	466	627	686	668	703	728
Female	99	90	92	102	92	114	124	99	126	179	154	148	117
Female %	18.9	17.5	17.7	19.5	17.4	21.3	20.1	17.5	16.7	20.7	18.7	17.4	13.8
Non-octogenarians	477	473	468	470	474	463	532	488	648	725	677	704	699
Octogenarians	48	40	53	53	55	72	84	77	105	140	145	147	146
Octogenarians %	9.1	7.8	10.2	10.1	10.4	13.5	13.6	13.6	13.9	16.2	17.6	17.3	17.3

## Data Availability

The data presented in this study are available on request from the corresponding author. The data are not publicly available due to ethical restrictions.
